# Antitumor Activities of Metal Oxide Nanoparticles

**DOI:** 10.3390/nano5021004

**Published:** 2015-06-09

**Authors:** Maria Pilar Vinardell, Montserrat Mitjans

**Affiliations:** Physiology Department, Faculty of Pharmacy, Universitat de Barcelona, Av. Joan XXIII s/n, 08028 Barcelona, Spain; E-Mail: montsemitjans@ub.edu

**Keywords:** antitumor, cancer therapy, metal oxide nanoparticles, *in vitro*, *in vivo*

## Abstract

Nanoparticles have received much attention recently due to their use in cancer therapy. Studies have shown that different metal oxide nanoparticles induce cytotoxicity in cancer cells, but not in normal cells. In some cases, such anticancer activity has been demonstrated to hold for the nanoparticle alone or in combination with different therapies, such as photocatalytic therapy or some anticancer drugs. Zinc oxide nanoparticles have been shown to have this activity alone or when loaded with an anticancer drug, such as doxorubicin. Other nanoparticles that show cytotoxic effects on cancer cells include cobalt oxide, iron oxide and copper oxide. The antitumor mechanism could work through the generation of reactive oxygen species or apoptosis and necrosis, among other possibilities. Here, we review the most significant antitumor results obtained with different metal oxide nanoparticles.

## 1. Introduction

Cancer is one of the principal causes of mortality worldwide and represents a serious health problem.

The last World Cancer Report of the World Health Organization states that the incidence of cancer increased from 12.7 million in 2008 to 14.1 million in 2012. This trend is projected to continue, with the number of new cases per year expected to rise a further 75% over the next two decades, which will bring the number of new cancer cases close to 25 million per year [1].

The treatment of cancer involves different therapies based on alkylating agents, antimetabolites, biological agents, *etc.*; but one of the principal problems is the side effects due to difficulties in differentiating between cancerous and normal cells, which produces systemic toxicity [2].

When exploring new strategies for the treatment of cancer, one possibility is the use of nanomaterials. For more than 30 years, nanomaterials have been used as pharmaceutical carriers to enhance the *in vivo* antitumor efficacy of drugs. The first studies in the 1970s used nanoscale drug carriers, such as liposomes entrapping antitumor pharmaceuticals.

The development of nanostructured devices for drug delivery and controlled release constituted new antitumor chemotherapies [3]. Recently, a systematic review was conducted in order to retrieve studies of the therapeutic effects of nanoparticles loaded with chemotherapeutic agents in digestive cancer tumors [4].

There is, however, a growing field of research into the use of nanoparticles (NPs) against tumor formation, development and progression, due to their intrinsic antitumor effects. The physicochemical properties that give NPs their anticancer activity are either related to intrinsic features, such as their antioxidant action, or depend on activities based on the application of external stimuli, such as hyperthermia in response to the application of infrared rays or magnetic fields. NPs with such properties may be used to play different roles in therapeutic antitumor approaches in photodynamic therapy and in hyperthermia. NPs stimulated by an external radiation source produce free radicals that kill cancer cells. In the case of radiotherapy, NPs may act as co-adjuvant agents, increasing the cell killing effect of ionizing radiation specifically on tumor cells. NPs may also act on the tumor environment, such as blood vessels or stroma, to reduce the development of tumor mass. Finally, NPs have been demonstrated to reduce the rate of tumor progression as a consequence of their antioxidant capacities [5].

The efficient carrier properties of NPs have enhanced their use in cancer treatment. NPs can be used to treat cancer by either passive or active processes. A passive process takes advantage of the enhanced permeability and retention (EPR) effect. The leaky vasculature found in cancerous tissue enables NPs to diffuse easily into the cancerous tissue and kill cells [6]. There are some side effects associated with drug delivery through passive processes. The leaky vasculature found in the cancerous cell is also found in inflamed tissue; as a result, there can be no strictly targeted drug delivery. The side effects this leads to can be reduced by targeting the drug through active processes. In an active process, the NPs are functionalized and targeted at the cancerous cell. Binding biomolecules or a ligand as a receptor to NPs can increase delivery to the targeted cancerous cells instead of normal cells. In anticancer therapy research, metal oxide NPs are used experimentally to directly kill tumor cells *in vitro* and *in vivo*. The results obtained with different metal oxide NPs are presented in this review, and their mechanisms of action are considered.

### 1.1. Iron Oxide

The tumor cell can be directly killed by non-toxic wavelength radiation, such as near-infrared (NIR), or oscillating magnetic fields (MF) that can be absorbed and transformed into toxic stimuli of reactive oxygen species (ROS) production or hyperthermia by NPs of iron oxide [7]. The advantage of this approach is derived from the fact that NPs, due to their particulate (as opposed to molecular) nature, can easily be directed to the tumor site by covalently linking tissue-specific molecular determinants or, in the case of magnetic NPs, with the aid of the external application of local magnetic fields. Therefore, such NPs can be used to selectively kill cancer cells, transforming radiant energy into heat or ROS. This helps reduce the damage to healthy tissues, which is among the most dangerous side effects of cancer therapy.

The use of magneto-sensitive NP complexes comprised of NPs of iron oxide and the anticancer drug doxorubicin increases the antitumor effect compared to conventional doxorubicin therapy. This increased antitumor effect is probably due to the fact that an external magnetic field can induce electron transitions in the nanocomplexes. The magneto complexes have additional free radicals, and doxorubicin acquires the magnetic properties of paramagnetic substances. The antitumor drug action is based on the activation of the hydroxyl radicals, which break mitochondria, lipids, proteins, DNA and other structures in tumor cells, and finally leads to their apoptosis or necrosis. The combined effect of a constant magnetic field and moderate radio frequency-induced hyperthermia (below 40 °C) and a magneto-sensitive nanocomplex of iron oxide and paramagnetic doxorubicin increased antitumor and antimetastatic action, compared to conventional diamagnetic doxorubicin. The synergy of magnetic fields and an anticancer magneto-sensitive nanocomplex provides a new strategy for future effective treatments of cancer [8].

Spherical iron oxide NPs were approved in the EU as a medical device for magnetic tumor hyperthermia in brain [9,10] and prostate cancer [11], in combination with radiotherapy or chemotherapy [12]. Recently, it has been observed that heat-generating PEG-coated iron oxide nanocubes interfere with the extracellular matrix of a tumor and have the potential to destructure the matrix under magnetic stimuli. This results in NP redistribution in the tumor during the three-cycle heating procedure, which also leads to the diminution of tumor growth [13].

Anticancer hyperthermia therapy consists in the application of heat at a temperature above 40 °C with the aim of killing tumor cells [14]. Different nanostructures have been used in hyperthermia applications, including magnetic iron oxide NPs. Once these nanomaterials are delivered to the tumor site, they efficiently absorb energy from an extrinsic source (e.g., NIR radiation or magnetic fields), transforming it into heat. Very promising results in hyperthermia treatments were obtained with magnetic iron oxide NPs, both in cell models and *in vivo*, as previously extensively reviewed [6]. When iron oxide NPs are exposed to radiofrequency oscillating magnetic fields, they produce heat due to the reorientation of the magnetization process.

The magnetization of super-paramagnetic iron oxide NPs (SPIONs) disappears as soon as the magnetic field is removed [15]. This is very important, because magnetization induces NP aggregation: a phenomenon that is acceptable during the treatment, but very dangerous to the patient after the treatment, when large aggregates may hinder the clearance of NPs and create serious health hazards. For this reason, for *in vivo* hyperthermia applications, the iron oxide NPs must be below the 30–40 nm size limit, to avoid the formation of ferromagnetic NPs. An increase in particle size will lead to higher saturation magnetization values and better performance for magnetic hyperthermia applications. However, this is only true when the particle is smaller than a critical size above which magnetic NPs become ferromagnetic (the so-called super-paramagnetic limit), which, in principle, is undesired magnetic behavior for biomedical applications, due to potential particle aggregation. Meanwhile, particle size is an issue of crucial interest in many biomedical applications in which the use of very small particles is highly desired, so that they can act as heat nano-sources in tumor regions of limited size access [16].

**Figure 1:**
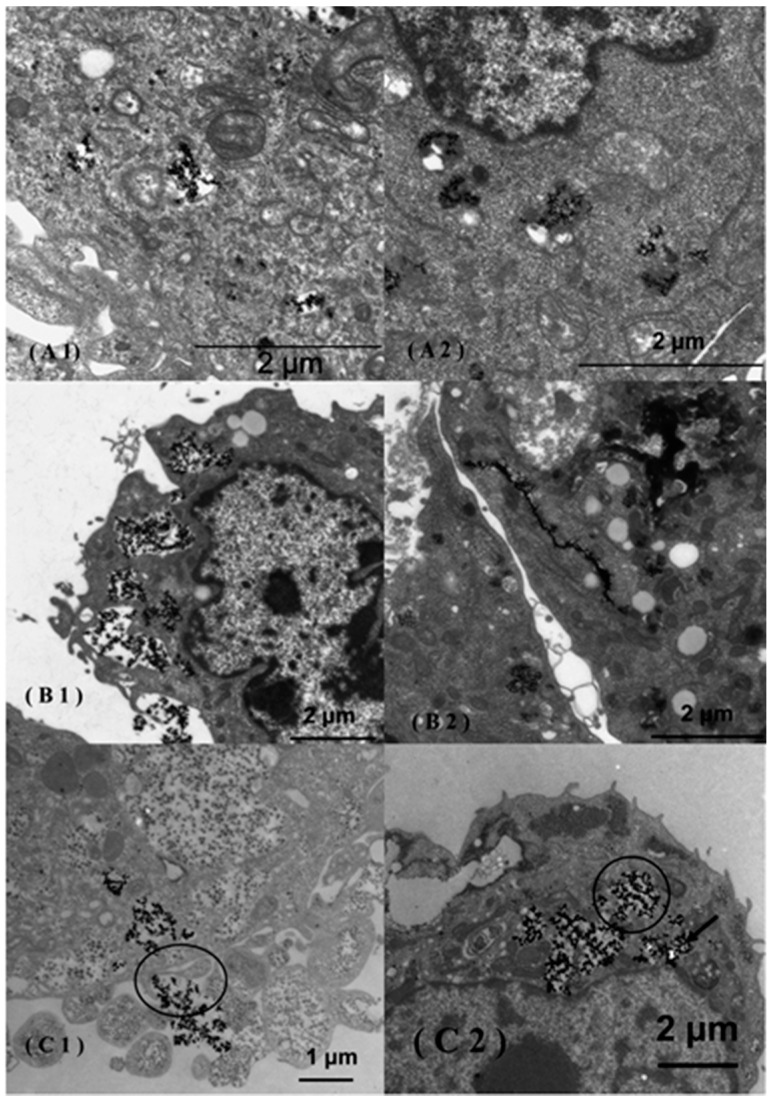
Transmission electron microscopy (TEM) images of MCF-7 (**A1**,**A2**), 3T3 (**B1**,**B2**) and Caco-2 (**C1**,**C2**) cells incubated in the presence of uncoated super-paramagnetic iron oxide NPs (SPIONs). The uncoated SPIONs were internalized via endocytosis in the Caco-2 cells (**C1**) and afterward released into the cytoplasm (**C2**). Citrate-coated SPIONs agglomerated in the cytoplasm of MCF-7 cells (**A2**) and were adsorbed along the endoplasmic reticulum in 3T3 cells (**B2**). Reproduced with permission from [17]. Copyright 2014, ACS Publications.

Recently, iron oxide NPs were studied as radio-sensitizing agents when using X-ray sources. *In vitro* studies showed that citrate- and malate-coated SPIONs sensitized tumor cells to X-rays by catalyzing ROS formation [17]. The results are promising and deserve further analysis of the biological mechanisms occurring downstream of the ROS production (Figure 1).

### 1.2. Titanium Dioxide

The principle behind photodynamic therapy (PDT) is that a photo-sensitizer (PS) consisting of a hydrophobic organic molecule is excited with electromagnetic radiation in the range of visible or UV light to generate cytotoxic ROS that induce apoptosis. The earliest photodynamic therapy protocols made use of polymeric and inorganic NPs as passive carriers to improve the solubility of the hydrophobic PS molecules and as platforms for their delivery to the tumor site [18]. An alternative approach consists of the use of inorganic NPs, such as titanium oxide (TiO_2_) [19,20,21], as direct photo-sensitizing agents that can therefore be used in place of PS molecules.

Photocatalyzed titanium dioxide (TiO_2_) NPs have been shown to eradicate cancer cells. However, the required *in situ* introduction of UV light limits the use of such therapy in humans. One strategy to overcome the limitations is the surface-functionalization of TiO_2_. Cell viability was observed to depend on particle concentrations, cell types and surface chemistry. Specifically, –NH_2_ and –OH groups showed significantly higher toxicity than –COOH. The results suggest that functionalized TiO_2_, and presumably other NPs, can be surface-engineered for targeted cancer therapy. In cancerous cell lines of T-24, HeLa and U937 cells, TiO_2_ particles were found to become incorporated into the cell membrane and the cytoplasm [20].

TiO_2_ NPs can be maintained for a long time in the body, and they are nontoxic and stable without light irradiation. A major challenge in photodynamic therapy is the direct illumination of the tissue by UV or visible light to trigger the TiO_2_ NPs. However, UV and visible light has a limited penetration distance in tissue, and this is a disadvantage when cancer cells are located far from the surface [22]. The maximum penetration into tissue can be obtained in the near-infrared (NIR) range (700–1000 nm), and this can be used for TiO_2_ NPs. Recently, the efficacy of NIR on crystallized shells composed of TiO_2_ NPs coated on the surface of NaYF4:Yb3þ,Tm3þ@NaGdF4:Yb3þ cores to form (NaYF4:Yb3þ, Tm3þ@NaGdF4:Yb3þ)@TiO_2_ core/shell nanocomposites (denoted as UCNPs@TiO_2_ NCs) has been demonstrated. Considering the deeper tissue penetration of NIR light than UV light, UCNPs@TiO_2_-based NIR light-mediated PDT possesses more effective tumor inhibition in comparison with UV light-irradiated UCNPs@TiO_2_. These effects have been demonstrated on HeLa cells *in vitro* and *in vivo* in a tumor model using female Balb/c nude mice [23].

Other authors have reported the synthesis of core-shell upconversion nanoparticles (UCNs) with a thin and continuous layer of TiO2, as nanotransducers with the ability to convert low-energy NIR light to high energy, which can penetrate deeper into the tissue [24]. The same authors have reported recently the capacity of these synthesized UCNs to reduce the production of tumors *in vivo* developed by injection of oral squamous cell carcinoma (OSCC) [25].

### 1.3. Cerium Oxide Nanoparticles

Cerium oxide NPs (CNPs) are a novel and very interesting material for radiation therapy, possessing the “smart” capacity to selectively induce the death of irradiated cancer cells [26], while protecting the surrounding tissue from radiation-induced damage and oxidative stress. Therefore, CNPs have the unique feature of acting as radio-protecting, as well as radio-sensitizing agents simultaneously. These NPs selectively increased oxidative stress and apoptosis in irradiated cancer cells, while protecting normal tissues [27,28]. It has been hypothesized that the selective toxicity of CNPs against cancer cells is due to the inhibition of the catalase-like activity of CNPs that occurs in acidic (pH 4.3) environments: in the presence of superoxide produced by the ionizing radiation, the SOD-like activity, which is maintained even at low pH, would lead to H_2_O_2_ accumulation, incrementing radiation toxicity [26]. The hypothesis is based on the assumption that cancer cells are acidic. In fact, the cytosol of cancer cells is slightly more alkaline (pH > 7.4) than normal cells; whereas the extracellular tumor microenvironment is slightly acidic due to the Warburg effect, and the pH decreases from 7.1 (normal tissues) down to 6.7 [29].

Another possible mechanism for the differential toxicity of CNPs on cancer cells would involve their SOD-like activity. It is known from many studies that the SOD enzyme acts as a radio-sensitizing agent, potentiating the DNA damage response and delaying the G2/M transition, thus favoring cell accumulation in G2 and allowing more thorough DNA repair. Therefore, CNPs may act as radio-sensitizing agents through an additional, biological mechanism that controls the response to DNA damage [30].

Recent results show that CNPs are almost as toxic as radiotherapy to the pancreatic cancer cells L3.6pl, but showed little or no toxic effect on the normal cells hTERT-HPNE. This finding points to the use of these NPs as a stand-alone therapy for pancreatic cancer treatment [26].

A recent study compared the effect of CNPs on cancer and normal human cells and concluded that the toxicity of these NPs is specific to the cancer cells. The low inhibitory potential of cerium oxide in normal human cell lines indicates that they may be safer for human usage in industry and medicine [31].

Other studies have shown that redox-active CNPs exhibit cytotoxic and anti-invasive effects on several cancer cells and are capable of sensitizing tumor cells to radiation, while protecting the normal cells in the stroma surrounding a tumor [32]. In squamous cell carcinoma of the skin and melanoma, CNPs exhibit pro-apoptotic and anti-invasive effects in a ROS-dependent manner. In contrast to conventional chemotherapeutics, CNPs are nontoxic to healthy, stromal cells of the skin. It has been reported that CNPs exert either pro-oxidant or antioxidant redox activity. Although CNP treatment increases the ROS level in tumor cells, resulting in apoptosis, CNPs showed antioxidant and protective properties in normal cells [33]. A medical application of CNPs may provide a promising possibility for skin cancer therapy and may be a valuable tool to supplement classical chemotherapeutics, such as doxorubicin, and protect against doxorubicin-induced cytotoxicity. Another advantage of CNPs is that they do not present genotoxic effects [34]. These results with CNPs of 5 nm are in contrast to another recent study with CNPs with a size of 16–22 nm, where DNA damaging effects were found in other tumor cell lines [32], indicating that the mode of action of the nanoparticles is strongly dependent on the size and cell type. The supplementation of conventional chemotherapies with CNPs may offer a novel strategy in the treatment of cancer, with greater benefit for patients, by enhancing antitumor activity and lowering the damaging side effects [34].

Previous studies were done *in vitro*, and it is necessary to demonstrate *in vivo* the efficacy of nanoparticles in the treatment of tumors. In this sense, *in vivo* xenograft studies with immunodeficient nude mice showed a decrease of tumor weight and volume after treatment with CNPs. This effect could be attributed to redox-active CNPs, which present selective pro-oxidative and antioxidant properties. This study was the first to show that CNPs prevent tumor growth *in vivo* [35].

### 1.4. Zinc Oxide Nanoparticles

Application of ZnO NPs, has shown that they are most efficacious on T98G cancer cells, moderately effective on KB cells and least toxic on normal human HEK cells. These results demonstrated that treatment with ZnO NPs sensitizes T98G cells by increasing both mitotic (linked to cytogenetic damage) and interphase (apoptosis) death. The ZnO NPs behave as genotoxic drugs, since they induce micronucleus formation in cells. These results could be helpful in designing more potent anticancer agents for therapeutic uses [36].

The apoptosis-correlated, intracellular production of ROS was also measured with melanoma cancer cells with varying ZnO NP doses [37].

**Figure 2:**
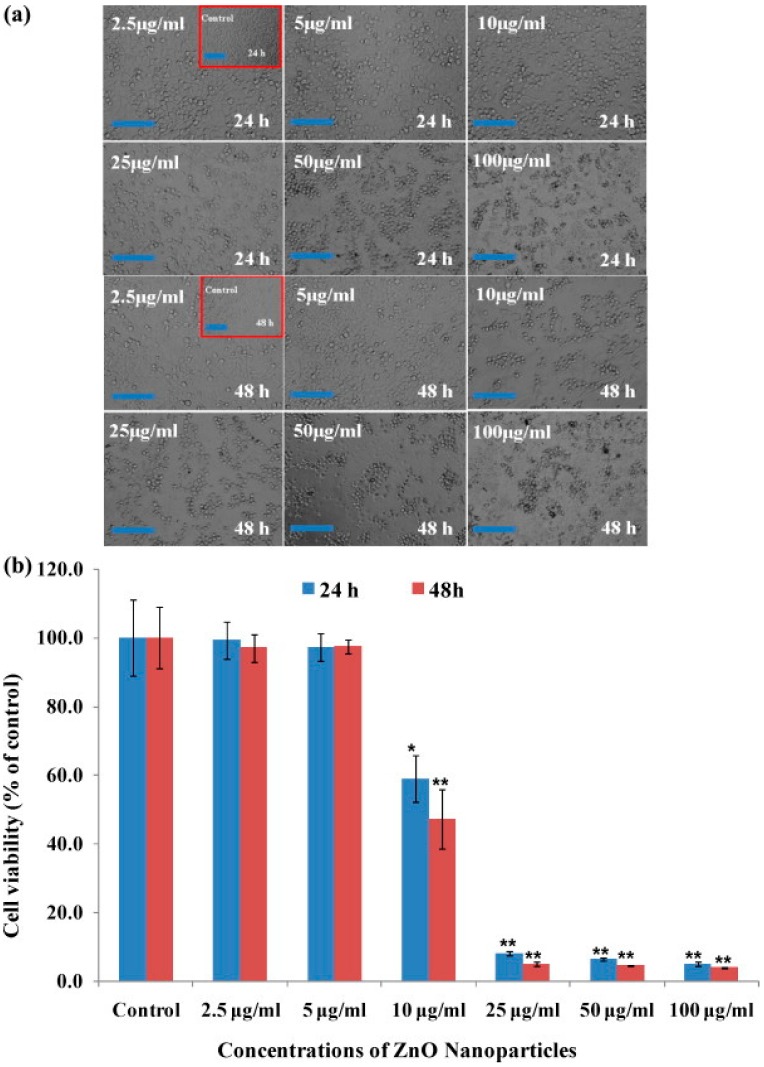
Microscopic images of HepG2 cells after treatment with ZnO NPs and the mitochondrial activity of cells exposed to various concentration of ZnO NPs for 24 and 48 h. The values are the mean ± SD of three independent experiments. * *p* < 0.01 and ** *p* < 0.001 *vs.* the control group (each scale bar = 1 mm). Reproduced with permission from [38]. Copyright 2014, Elsevier.

Zinc oxide NPs were used at a very low concentration and were found to exhibit activity against HepG2 (liver cancer) and MCF-7 (breast cancer) cancer cells in a dose-dependent manner: viability, measured by the MTT assay, showed a dose-dependent decrease. At a very low concentration, such as 25 µg/mL, the cell viability was less than 10% in the case of HepG2 cells (Figure 2). The results of these anti-proliferative studies clearly demonstrate that treatments with NPs sensitize cancer cells. The degree of apoptosis was found to be enhanced with an increase in the concentration of NPs, and a significant concentration of NPs resulted in cell death in both cancer cell lines [38]. In that study, quantitative real-time PCR was utilized to analyze the mRNA levels of apoptotic markers (p53, Bax, bcl-2 and caspase-3) in HepG2 cells exposed to ZnO NPs at a concentration of 50 μg/mL for 24 h. The results showed that the mRNA levels of these apoptotic markers were significantly altered in HepG2 cells due to ZnO NP exposure. The mRNA level of the tumor suppression gene p53 was 1.9-fold higher and the mRNA expression levels of the pro-apoptotic gene Bax and the anti-apoptotic gene bcl-2 were decreased (2.7- and 2.5-fold, respectively) in the exposed cells, compared to untreated cells. Moreover, the effect of ZnO NPs on the mRNA expression level of caspase-3 was studied and was found to be 1.8-fold higher in the treated cells than the untreated control cells. The mRNA expression levels of p53, Bax, bcl-2 and caspase in HepG2 cells in response to ZnO NP exposure was studied, because apoptosis is controlled through these pathways. The quantitative real-time PCR results show that ZnO NPs upregulate the mRNA levels of the cell cycle checkpoint protein p53 and the pro-apoptotic protein Bax. The expression of the anti-apoptotic protein bcl-2 was downregulated in cells exposed to ZnO NPs. Furthermore, the upregulation of p53 and the downregulation of bcl-2 family members, such as Bax, induce the permeabilization of the outer mitochondrial membrane, which releases soluble proteins from the intermembrane space into the cytosol, where they promote caspase activation [38].

**Figure 3:**
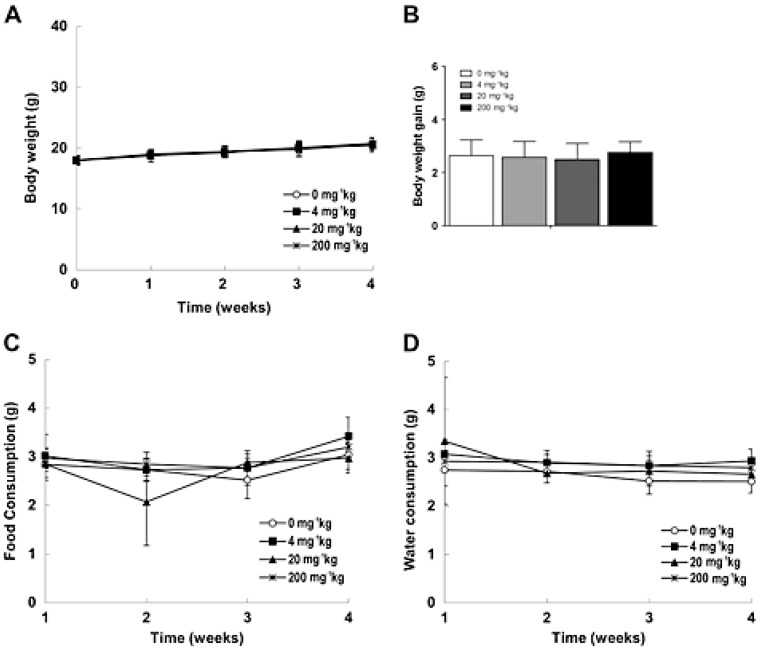
Body weight (**A**), body weight gain (**B**), food (**C**), and water (**D**) consumption in mice injected with core-shell NPs (CSNPs) for four weeks. Reproduced with permission from [42]. Copyright 2015, John Wiley & Sons, Ltd.

Therapeutic cancer vaccines are emerging as part of an anticancer regimen that utilizes specific antigens to initiate and modulate the antitumor immune response [39]. Dendritic cells (DCs) have been used for therapeutic cancer vaccines [40]. DC-based cancer immunotherapy that destroys tumors requires a clinically-suitable delivery system for the target antigens in the DCs. In one study, the authors developed iron oxide (Fe_3_O_4_)-zinc oxide (ZnO) core-shell NPs (CSNPs) to have ZnO-binding peptides to carry tumor antigens in DCs. This NP-antigen complex was efficiently taken up by the DCs and was demonstrated to function as a cancer immunotherapy via injection of the DCs containing the CSNP-antigen complex into the hind footpads of mice. Mice immunized with DCs containing the NP-antigen complex showed enhanced tumor antigen-specific T-cell responses, delayed tumor growth and better survival than controls [41]. However, a study of the potential toxicity of these new nanocarriers is required. Recently, a repeated toxicity study was performed *in vivo* by subcutaneous injection in mice and showed a dose-dependent increase in granulomatous inflammation at the injection site of the CSNP-treated animals, but no alterations in the body (Figure 3) and other histopathological lesions in other organs could be attributed to the CSNPs [42].

The electrostatic properties of zinc oxide determine that it can have different charges on its surface under acid and base conditions. This can be used in the conjugation of therapeutic agents and also to internalize NPs within cancer cells, as they are high in phospholipids with negative charges on their surface. Zinc oxide NPs also have a photodynamic property: illumination leads to the production of large amounts of ROS and can result in cell apoptosis [43]. These qualities make zinc oxide NPs a suitable candidate for a drug carrier. Conjugation of therapeutic agents with zinc oxide NPs may yield better results than with other NPs, in the targeted cancerous cells instead of normal cells.

The decrease in cell viability of MCF-7 cell lines after treatment with a metalloprotein confirms that synthesized metalloproteins can have an anticancer property. NPs carrying asparaginase were found to be more specific, highly effective and to provide reliable results [44].

ZnO NP pre-incubation followed by UVA-1 irradiation induced a significant reduction in viable head and neck squamous cell carcinoma cell lines (HNSCC) *in vitro* [45].

One strategy to reduce the toxicity of typical cancer therapies is their combination with NPs. The combination of ZnO NPs with paclitaxel and cisplatin [46] or daunorubicin [47] increases the effect of these chemotherapeutics in cell lines *in vitro*. It is evident that differently-sized ZnO NPs could greatly facilitate drug targeting and accumulation of daunorubicin in leukemia cancer cells and could thus act as an efficient agent to enhance drug delivery [47].

The combination of NPs with typical cancer drugs allows the reduction of the drug dose with the corresponding reduction in side effects.

### 1.5. Copper Oxide Nanoparticles

Studies report the biosynthesis of copper oxide NPs (CONPs) from different plant extracts, such as that of *Ficus religiosa* [48] or *Acalypha indica* [49]. These NPs showed cytotoxic effects on A549 human lung cancer cells and MCF-7 breast cancer cells, respectively. The mechanism of cytotoxicity was demonstrated to be through the induction of apoptosis with enhanced ROS generation. The green synthesis of these NPs has been proposed as a reliable, simple, nontoxic and eco-friendly method [50]. CONPs have many industrial applications [51], but recent studies have reported the antifungal and bacteriostatic properties of copper NPs/polymer composites [52].

**Figure 4:**
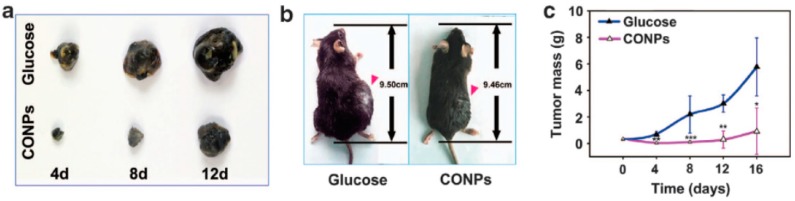
Antitumor effects of copper oxide NP (CONP) therapy on subcutaneous melanoma and metastatic lung tumors. (**a**) Representative images of stripped subcutaneous tumors; (**b**) Representative images of mice bearing subcutaneous melanoma from the same study at Day 12; (**c**) Plot of tumor mass *versus* time. Reproduced with permission from [54]. Copyright 2015, Associazione Differenziamento e Morte Cellulare.

*In vitro* studies demonstrated that cuprous oxide nanoparticles (CONPs) selectively induce the apoptosis of tumor cells *in vitro* [53]. Thereafter, the same authors studied the antitumor properties of CONPs *in vivo*, using the particles to treat mouse subcutaneous melanoma and metastatic lung tumors, based on B16-F10 mouse melanoma cells, by intratumoral and systemic injections, respectively. The results showed that CONPs significantly reduced the growth of melanoma, inhibited the metastasis of B16-F10 cells and increased the survival rate of tumor-bearing mice. The subcutaneous tumors of the CONP group were clearly smaller than those observed in the glucose control group (Figure 4). To explore the clearance of CONPs, mice were injected with particles via the vena caudalis at a dose of 2 mg/kg, and after seven days, the major organs were harvested and observed. Importantly, the results also indicated that CONPs were rapidly cleared from the organs and that the particles exhibited little systemic toxicity.

Furthermore, the authors observed that CONPs targeted the mitochondria of HeLa cells *in vitro*, which resulted in the release of cytochrome C from the mitochondria and the activation of caspase-3 and caspase-9 after the CONPs entered the cells. In conclusion, CONPs can induce the apoptosis of cancer cells through a mitochondrion-mediated apoptosis pathway, which raises the possibility that CONPs could be used to cure melanoma and other cancers. CONPs inhibit the growth and metastasis of melanoma in a tumor-bearing mice model and are rapidly cleared by mice with low toxicity [54].

CONPs were found to induce cytotoxicity in a human liver carcinoma cell line (HepG2) in a dose-dependent manner, which was probably mediated through ROS generation and oxidative stress [55].

### 1.6. Silicon Dioxide or Silica

In contrast to other metal oxide NPs with antitumor effects *per se*, silica has been shown to be a good carrier for different anticancer drugs, such as gemcitabine and paclitaxel, in the treatment of pancreas cancer in mice [56].

The size- and shape-controllable pores of mesoporous silica NPs (SNPs) can store pharmaceutical drugs and prevent their premature release and degradation before reaching their designated target. Chemotherapeutic drugs can be loaded into mesoporous silica NPs, replacing the need to use solvents that are often toxic for healthy tissues.

The first concern is the potential toxicity of this kind of NP; so it is necessary to study subacute toxicity with mesoporous SNPs. This study showed no toxicity after *in vivo* administration to mice [57]. Thereafter, the authors reported the design, synthesis and biological evaluation of surface-modified SNPs for the delivery of camptothecin (CPT). The tumors in the mice treated with NPs loaded with CPT were virtually eliminated at the end of the experiments. These results proved that the high drug-loading capacity, low toxicity and tumor-accumulating effect of these NPs provide a promising drug delivery vehicle for anticancer drugs. The tumor-curing results were also encouraging, constructing a base for further research into the capacity of MSNs for other types of tumors and the possibility of using lower dosages to further reduce toxicity. SNPs have a natural tendency to aggregate, due to hydrogen-bond interactions between external silanol groups. The use of this type of nanomaterial in biological applications requires the reduction of this natural trend, in order to improve biodistribution and cellular uptake. Coating the NP surface with trihydroxysilylpropyl carboxylate (THSC) groups has a considerable effect on the surface charge of SNPs (−1.8 and −13.9 mV for non-coated SNP and SNP-COOH, respectively) with the electrostatic repulsion being enough to reduce aggregation and to increase the stability of the particles in aqueous solution [58]. A drug has been covalently linked to the NP through an ester bond with the 20-hydroxy moiety, in order to stabilize its lactone ring and to avoid unspecific release of the drug. The material obtained is highly stable in plasma, with slow release of the cargo at physiological pH values. Cell internalization and *in vitro* efficacy assays demonstrated that SNPs carrying CPT entered cells via endocytosis, and the intracellular release of the cargo induced cell death with half maximal inhibitory concentration (IC_50_) values and cell cycle distribution profiles similar to those observed for the naked drug. Furthermore, the *in vivo* biodistribution, therapeutic efficacy and biocompatibility of the SNP-CPT were evaluated in human colorectal cancer xenografts using *in vivo* fluorescence or bioluminescence optical imaging. *In vivo* tumor-accumulation and whole-body tissue distribution were studied based on the acquisition of fluorescence emission of a fluorophore (Cy5.5) conjugated to the SNP-CPT, as well as by HPLC quantification of tissue CPT levels. The results showed that although the SNP-CPT tended to accumulate in organs of the reticuloendothelial system, the SNPs boost CPT concentration in tumor compared to administration of the free drug. Accordingly, SNP-CPT treatment delayed the growth of subcutaneous tumors while significantly reducing the systemic toxicity associated with CPT administration. These results indicate that SNP-CPT could be used as a robust drug delivery system for antitumor treatments based on CPT [59]. The authors indicated that although their results regarding tumor growth differences were not as dramatic as those obtained previously by Lu *et al.* [57], a distinct type of experiment was carried out in each case. Botella *et al.* [58] performed a tumor growth inhibition experiment starting treatment once the tumors were well established; in contrast, Lu *et al.* performed a tumor growth prevention assay, and the therapeutic agents were also administered at higher doses via a different route [59].

### 1.7. Influence of Size and Characteristics of Nanoparticles

Different strategies have been developed for cancer treatment; the first exploits the enhanced permeation and retention effect (EPR) associated with the hyperpermeability of the tumor vasculature where sufficiently small particles (<500 nm) can passively extravasate and accumulate in tumor parenchyma [60].

The size of the different metal oxide nanoparticles appears in Table 1 and Table 2, where we can observe that in most cases, the particles are smaller than 50 nm. The measures of the NPs are usually performed in water, but when NPs are in cell culture medium, aggregates are observed. This is one of the principal limitations in knowing the real size of NPs when *in vitro* studies are performed.

**Table 1:** Metal oxide NPs with anticancer activity by direct action on tumor cells in different *in vivo* models.

Metal Oxide NPs	*In Vivo* Model	NP Size	Authors	Reference
Cerium oxide	Orthotopic injection of pancreatic cancer cells in athymic nude mice	5–8 nm	Wason *et al.*, 2013	[26]
Cerium oxide	Xenografted mice injected with melanoma cells	3–5 nm	Ailili *et al.*, 2013	[35]
Titanium dioxide	Human oral squamous cell carcinoma tumor in mice	50 nm	Lucky *et al.*, 2015	[25]
Copper oxide	Subcutaneous melanoma in mice	40–110 nm	Wang *et al.*, 2013	[54]

The uptake, localization and effect of cerium oxide nanoparticles in the cells depend on their size, surface charge and agglomeration inside the cells. Particles of a diameter less than 20 nm are present longer in the cells than larger particles. The uptake of single, unagglomerated nanoparticles therefore becomes very improbable for particles of less than 50 nm in size; then, particle size is indirectly the dominant factor determining the rate of uptake, while primary particle number concentration and total surface area are of minor importance [31,61].

Studies reporting the use of TiO2 in photodynamic therapy have presented a nonuniform size of the nanoconstruct that might have compromised repeatability and translatability of photodynamic therapy results *in vivo* [25].

When NPs are administered *in vivo* and are present in blood, then protein corona can be formed, modifying their action. NP-protein coronas generally reduce cytotoxicity and immunotoxicity, but immunotoxicity can be mitigated or activated depending on the type of NP and adsorbed plasma protein. At this time, there is limited knowledge of the correlation between the physicochemical properties of NPs and their physiological effects. The formation and immunological response to NP-protein coronas is significantly influenced by the physiochemical surface properties of the NPs (*i.e.*, physical surface architecture and chemical functionality), and thus, future works should address this by analyzing protein distribution and examining *in vitro* and *in vivo* responses [62].

**Table 2:** Metal oxide NPs with anticancer activity by direct action on tumor cells in different *in vitro* studies. HNSCC, head and neck squamous cell carcinoma.

Metal Oxide	*In Vitro* Model	NP Size	Organ Origin	Authors	Reference
Cerium oxide	Pancreatic cancer cell line L3.6pl	5–8 nm	Pancreas	Wason *et al.*, 2013	[26]
Cerium oxide	Non-small cell carcinoma NCI-H460 DLD1 and HT-29 adenocarcinoma	4 nm	Lung colon/rectum	Pešić *et al.*, 2015	[31]
Cerium oxide	A375 melanoma cells	5 nm	Skin	Sack *et al.*, 2014	[32]
Cerium oxide	Squamous carcinoma cell lines SCL-1	3–5 nm	Skin	Ailili *et al.*, 2013	[35]
Copper oxide	HeLa cells from human cancerous cervical tumor and B16-F10 melanoma cells from mice	40–110 nm	Cervix skin	Wang *et al.*, 2013	[54]
Copper oxide	HepG2 cells	22 nm	Liver	Sidiqqui *et al.*, 2013	[54]
Copper oxide	A549 adenocarcinomic human alveolar basal epithelial cells	577 nm	Lung	Sankar *et al.*, 2014	[48]
Iron oxide	Caco-2 cells MCF-7 cells	9–20 nm	Colon breast	Klein *et al.*, 2014	[17]
Titanium oxide	Human bladder cancer cell line T24, HeLa and human myeloid leukemia cell line U937	21 nm	Bladder blood	Thevenot *et al.*, 2008	[20]
Zinc oxide	T98G human glioblastoma cells	13 nm	Brain	Wahab *et al.*, 2013a	[36]
Zinc oxide	HNSCC carcinoma cell lines	74 nm	Neck	Hackenbert *et al.*, 2010	[45]

## 2. Conclusions

Different strategies use metal oxide NPs in the treatment of cancer. In some cases, the NP alone shows antitumor effects, both *in vitro* and *in vivo*, by direct action on tumor cells or indirectly via the action of heat.

In other cases, the NPs could facilitate the action of a typical anticancer drug, reducing the dose required and the side effects that are the principal disadvantage of such drugs.

Several studies demonstrated the antitumor activity of different metal oxide nanomaterials *in vitro* and only a few studies *in vivo*; the latter showing the action of cerium oxide (CNPs) and cuprous oxide (CONPs) nanoparticles. Recently, the effect of titanium dioxide (TiO_2_) stably coated on individual UCN cores with the application of NIR to penetrate tumors of OSCC induced in mice has been demonstrated.

In conclusion, there has been an increase in recent years in the research of the potential use of metal oxide NPs for cancer treatment. However, there are still limitations due to the heterogeneity of the cell used for each tumor model *in vitro* and/or *in vivo*, which make it difficult to do a comparison between the different studies. Another limitation is the formation of protein corona when NPs reach the blood and interact with the plasma proteins, affecting *in vivo* distribution and clearance. Research continues in this area, and more information about the distribution, biocompatibility and low toxicity for normal tissues is necessary prior to clinical trials.
